# Iron corrosion concomitant with nitrate reduction by *Iodidimonas nitroreducens* sp. nov. isolated from iodide-rich brine associated with natural gas

**DOI:** 10.3389/fmicb.2023.1232866

**Published:** 2023-09-22

**Authors:** Takao Iino, Kenshiro Oshima, Masahira Hattori, Moriya Ohkuma, Seigo Amachi

**Affiliations:** ^1^Japan Collection of Microorganisms (JCM), RIKEN BioResource Research Center (RIKEN-BRC), Tsukuba, Japan; ^2^Center for Omics and Bioinformatics, Graduate School of Frontier Sciences, The University of Tokyo, Kashiwa, Japan; ^3^Graduate School of Horticulture, Chiba University, Matsudo, Japan

**Keywords:** microbially influenced corrosion, iron corrosion, nitrate-reduction, iodide oxidation, *Iodidimonas*

## Abstract

Microbially influenced corrosion (MIC) may contribute significantly to corrosion-related failures in injection wells and iron pipes of iodine production facilities. In this study, the iron (Fe^0^) corroding activity of strain Q-1 isolated from iodide-rich brine in Japan and two *Iodidimonas* strains phylogenetically related to strain Q-1 were investigated under various culture conditions. Under aerobic conditions, the Fe^0^ foil in the culture of strain Q-1 was oxidized in the presence of nitrate and yeast extract, while those of two *Iodidimonas* strains were not. The amount of oxidized iron in this culture was six times higher than in the aseptic control. Oxidation of Fe^0^ in aerobic cultures of nitrate-reducing bacterium Q-1 was dependent on the formation of nitrite from nitrate. This Fe^0^ corrosion by nitrate-reducing bacterium Q-1 started after initial nitrite accumulation by day 4. Nitrate reduction in strain Q-1 is a unique feature that distinguishes it from two known species of *Iodidimonas*. Nitrite accumulation was supported by the encoding of genes for nitrate reductase and the missing of genes for nitrite reduction to ammonia or nitrogen gas in its genome sequence. Phylogenetic position of strain Q-1 based on the 16S rRNA gene sequence was with less than 96.1% sequence similarity to two known *Iodidimonas* species, and digital DNA–DNA hybridization (dDDH) values of 17.2-19.3%, and average nucleotide identity (ANI) values of 73.4-73.7% distinguished strain Q-1 from two known species. In addition of nitrate reduction, the ability to hydrolyze aesculin and gelatin hydrolysis and cellular fatty acid profiles also distinguished strain Q-1 from two known species. Consequently, a new species, named *Iodidimonas nitroreducens* sp. nov., is proposed for the nitrate-reducing bacterium strain Q-1*^T^*.

## 1. Introduction

Iodine is an essential trace element for vertebrates because of its role as a component of thyroid hormones. Inadequate dietary iodine intake leads to iodine deficiency disorders such as endemic goiter and cretinism, which remain the leading causes of intellectual disability and brain damage worldwide (Hetzel, 1983; Hetzel and Mano, 1989). Iodine is also used commercially in the production of X-ray contrast media, antiseptics, pharmaceuticals, polarizing films and industrial catalysts. Molecular iodine is produced commercially from iodide ions in underground brine. At iodine production facilities, brine is extracted from a production well, and waste brine is injected into an underground aquifer through an injection well. However, injection wells and iron pipes are subject to severe corrosion and clogging. In particular, microbiologically influenced corrosion (MIC) is suspected to be a major cause of corrosion-related failures due to biomass accumulation at the waste brine injection well (Lim et al., 2011; Wakai et al., 2014). Corrosion costs exceed 3% of the world’s GDP, and MIC may contribute significantly to the overall corrosion risk, especially in the gas and oil industry. In anaerobic environments, a specific group of sulfate-reducing bacteria (SRB) accelerates Fe^0^ corrosion, with recent evidence suggesting that direct electron transfer from Fe^0^ to SRB is mediated extracellularly via outer membrane cytochromes (Dinh et al., 2004; Venzlaff et al., 2013; Enning and Garrelfs, 2014; Beese-Vasbender et al., 2015; Deng et al., 2018). Some hydrogenotrophic methanogens that reduce carbon dioxide to methane also cause MIC on Fe^0^ by secreting specific [NiFe] hydrogenase that catalyzes Fe^0^ oxidation to ferrous ion: Fe^0^ + 2H^+^ → Fe^2+^ + H_2_ (Daniels et al., 1987; Mori et al., 2010; Uchiyama et al., 2010; Tsurumaru et al., 2018). In iodine production plants, denaturing gradient gel electrophoresis (DGGE) analysis and culture-dependent approaches revealed that iodide-oxidizing bacteria (IOB) were predominant in native brine samples containing carbon steel coupons, and the corrosion rate by four IOB isolates increased in the presence of potassium iodide (Wakai et al., 2014). However, the process of MIC by IOB is not yet understood, and further research is needed.

Iodide-oxidizing bacteria catalyze the oxidation of iodide to molecular iodine (I_2_). These bacteria were phylogenetically divided into two groups in the class *Alphaproteobacteria*; one group was closely related to the genus *Roseovarius*, while the other was the genus *Iodidimonas*, which was previously recognized as a unique lineage distantly related to the genus *Rhodothalassium* (Amachi et al., 2005; Iino et al., 2015). To date, more than 30 valid species are included in the genus *Roseovarius*, which mainly consists of non-phototrophic bacteria with polyhydroxybutyrate accumulation and bacteriochlorophyll a production (Pujalte et al., 2014). On the other hand, the genus *Iodidimonas* contains only two species so far: *Iodidimonas muriae* (Iino et al., 2016) and *Iodidimonas gelatinilytica* (Iino et al., 2021a). Therefore, the species diversity and phylogenetic diversity in this genus *Iodidimonas* are poorly understood compared to *Roseovarius*. A clear understanding of the phenotypic characterization and phylogenetic localization of Fe^0^-corroding microorganisms and the elucidation of the mechanism of MIC are increasingly important for effective MIC prevention and control, as genomic and metagenomic analyses are often applicable for rapid exploration of Fe^0^-corroding microorganisms inhabiting corrosive environments. In this study, the Fe^0^-corroding activity of *Iodidimonas* strains was investigated under various culture conditions, and the taxonomic status of strain Q-1 was also characterized as a new species during the course of this study.

## 2. Materials and methods

### 2.1. Bacterial strains and culture conditions

Iodide-oxidizing bacterium strain Q-1, isolated from iodide-rich brine associated with natural gas in Miyazaki, Japan (Amachi et al., 2005; Amachi and Iino, 2022), was used in this study. *I. muriae* strain C-3*^T^* (= JCM 17843*^T^* = LMG 28660*^T^*) (Iino et al., 2016) and *I. gelatinilytica* strain Hi-2*^T^* (= JCM 17844*^T^* = LMG 28661*^T^*) (Iino et al., 2021a) were also used in this study because these strains phylogenetically located near strain Q-1 (Amachi et al., 2005; Iino et al., 2016).

An artificial seawater medium containing 0.1% (wt/vol) yeast extract (YSw medium) was prepared according to the method described previously (Iino et al., 2015). The pH of the medium was adjusted to 7.0 with 6 N HCl, and 20 ml of the medium was added to each 70 ml serum bottle. Dissolved air was removed by flushing with N_2_-CO_2_ [4:1 (vol/vol)], and the bottles were sealed with butyl rubber stoppers (Nichiden-Rika Glass, Hyogo, Japan) and aluminum caps (Nichiden-Rika Glass, Hyogo, Japan). Oxygen/yeast extract/seawater (OYSw) medium was prepared by adding 2.5 ml oxygen to the bottle with YSw medium through a 0.2 μm pore size membrane filter. Prior to inoculation, 0.2 ml of vitamin solution (Wolin et al., 1963) was added to each bottle after filtering the solutions through a 0.2 μm pore size membrane filter (Millex-GV; Merck Millipore, MA, USA).

Strain Q-1, *I. muriae* C-3*^T^* and *I. gelatinilytica* strain Hi-2*^T^* were maintained in OYSw medium or on Marine Agar 2216 (BD, NJ, USA) at 30°C.

### 2.2. Growth tests with potential electron donors and acceptors

For the growth tests of each bacterial isolate, a pre-culture was prepared by growing an isolate in OYSw medium as described above at 30°C for 3 days. Then, 0.1 ml of the pre-culture was inoculated into 10 ml of YSw medium supplemented with different electron acceptors at 2–10 mM. The culture was then grown at 30°C for 28 days under either N_2_-CO_2_-O_2_ [14:5:1 (vol/vol/vol)] or N_2_-CO_2_ [4:1 (vol/vol)]. The resulting growth was determined by measuring turbidity at 660 nm using a spectrophotometer (model DU730; Beckman Coulter, CA, USA).

### 2.3. Fe^0^ corrosion test

The Fe^0^-corroding activities of *Iodidimonas* strains were tested in a corrosion test medium consisting of YSw medium supplemented with 100 mM HEPES buffer (pH 7.0) under an atmosphere of N_2_-CO_2_-O_2_ (15:4:1) or N_2_-CO_2_ (4:1). Fe^0^ foils (purity > 99.99%, 10 × 10 × 0.1 mm, approximately 80.0 mg) were purchased from Sigma-Aldrich. The Fe^0^ foil was placed in a 70 ml serum bottle; air was removed from the bottle by flushing with N_2_-CO_2_ [4:1 (vol/vol)], and the bottle was sealed with a butyl rubber stopper. The Fe^0^ foil was then sterilized in an autoclave at 121? for 15 min. prior to the Fe^0^ corrosion test. Twenty milliliters of medium were anaerobically and aseptically added to a 70 ml serum bottle containing the Fe^0^ foil, and 10 mM potassium iodide or 10 mM nitrate (final concentration) was added to the medium through a 0.2 μm pore size membrane filter. To prepare aerobic conditions, 2.5 ml oxygen [5% (vol/vol) at final concentration] was added to the bottle with corrosion test medium through a 0.2 μm pore size membrane filter. The initial oxygen concentration was kept at 5% (vol/vol) because iron was chemically oxidized in a large amount under air atmosphere. Then, 0.2 ml of a bacterial pre-culture with approximately 0.5 in terms of turbidity by reading the absorbance at 660 nm was added to the medium, and the culture was incubated at 30°C for 28 days.

### 2.4. Chemical analyses

After cultivation, 100 μl of culture fluids containing oxidized iron were recovered into microtubes, and acidified with 50 μl of 6 N HCl to dissolve precipitated iron in the culture fluids. The fluids were then added with 100 μl of 1 M ascorbic acid to reduce ferric ions to ferrous ions and to quantify total iron (ferrous and ferric ions). The iron ion concentration in each acidified solution was determined colorimetrically using *o*-phenanthroline as described by Sandell (1959). For the quantification of nitrate, nitrite, and ammonium ions, the culture fluids were centrifuged at 20,400 × *g* for 10 min. The supernatant was collected and filtered through a 0.2 μm pore size membrane filter. Iodide, nitrate, nitrite, and ammonium ions in the culture were quantified using a high-performance liquid chromatography (HPLC) system (model HIC-20Asuper; Shimadzu Corp., Kyoto, Japan) equipped with a conductivity detector (model CDD-10ADsp; Shimadzu Corp., Kyoto, Japan), a Shim-Pack cation column (IC-C4; Shimadzu Corp., Kyoto, Japan), and a Shim-Pack anion column (IC-SA2; Shimadzu Corp., Kyoto, Japan).

### 2.5. DNA extraction, genome sequencing, and phylogenetic and genome analyses

For draft genome sequencing, cells of strain Q-1 cultured on marine agar at 30°C for 3 days were harvested, and genomic DNA was extracted using a Genomic-tip 100/G kit (Qiagen, Hilden, Germany). Using Qubit 4 Fluorometer (Thermo Fisher Scientific, MA, USA), the quantity and quality of genomic DNA were confirmed that more than 100 μg of DNA with absorbance *A*260/*A*230 and *A*260/*A*280 more than 1.6 was recovered from cells of strain Q-1. Sequencing libraries of strain Q-1 was constructed using an Ion Xpress Plus Fragment Library Kit (Thermo Fisher Scientific, MA, USA), in accordance with the supplier’s protocol. Draft genome sequences were determined using an Ion Torrent PGM system and Ion PGM 200 Sequencing Kit (Thermo Fisher Scientific, MA, USA). A total of 697,549 reads were assembled into 60 contigs for strain Q-1 using Newbler version 2.8 (Roche). The assembled draft genome sequence was annotated using the RAST^1^ server version 2.0 (Aziz et al., 2008). Metabolic pathways were reconstructed by uploading Fasta files containing contigs of amino acid sequences to the KEGG automated annotation server (Moriya et al., 2007). After aligning the 16S rRNA gene sequences with an ARB software (Ludwig et al., 2004), phylogenetic trees were constructed using the neighbor-joining (NJ) method with a CLUSTAL X program (Saitou and Nei, 1987; Thompson et al., 1997) and the maximum-likelihood (ML) method with a MORPHY software version 2.3b3 (Felsenstein, 1981; Hasegawa et al., 1985). To calculate the overall genomic relatedness, digital DNA–DNA hybridization (dDDH) values were determined using formula 2 by uploading the Fasta files containing the contigs of the genome sequences or the amino acid sequences into a Genome–to–Genome Distance Calculator (GGDC) v 2.1 with BLAST+^2^ (Meier-Kothoff et al., 2013), and average nucleotide identity (ANI) values were determined using an ANI Calculator^3^ (Goris et al., 2007).

### 2.6. Morphological, biochemical and physiological characterization

Routine microscopic observation was performed using a phase-contrast microscope (Eclipse C*i*-L plus; Nikon, Tokyo, Japan) and an S4E stereomicroscope (Leica, Wetzlar, Germany). Morphology of bacterial cells was observed using a transmission electron microscope (TEM; JEM-1400plus; JOEL, Tokyo, Japan) operated at 100 kV. Cells were stained with 2% (wt/vol) phosphotungstic acid (pH 6.5–7.0). For ultramicrotomy, bacterial cells held with two copper grids were fixed by rapid immersion in liquid propane cooled at –175°C, and transferred to an ethanol solution containing 2% glutaraldehyde, 1% tannic acid, and 2% distilled water at –80°C. The samples were embedded in Quetol-812 (Nisshin EM Co., Tokyo, Japan), and ultrathin sections were cut with an Ultracut UCT (Leica, Wetzlar, Germany) equipped with a diamond knife. Sections on grids were stained by floating on single drops of 2% uranyl acetate–lead staining solution (Sigma-Aldrich Co., MO, USA), and observed by TEM (JEM-1400plus) at 100 kV. Conventional Gram staining was performed according to the method of Hucker and Conn (1923). Phenotypic characterization was performed as previously described (Iino et al., 2016). Iodide oxidation was determined by the formation of purple pigment on marine agar containing 1.0 g potassium iodide l^–1^ and 1.2 g soluble starch l^–1^, according to the method of Amachi et al. (2005). Photosynthetic growth was determined in YSw medium at a light intensity of 2,500 lux. Aesculin hydrolysis was determined by the formation of black pigment on marine agar containing 1.0 g aesculin l^–1^ and 0.5 g ferric citrate l^–1^, according to the method of Edberg et al. (1977). Gelatin liquefaction was determined by keeping at 4°C after cultivation at 30°C for 1 week on marine agar containing 120 g gelatin l^–1^, according to the method of Pickett et al. (1991).

### 2.7. Chemotaxonomic characterization

Cells of strain Q-1, *I. muriae* C-3*^T^*, and *I. gelatinilytica* Hi-2*^T^* cultured on marine agar at 30°C for 3 days were harvested to determine their chemotaxonomic properties. Isoprenoid quinones were determined using the HPLC method described by Komagata and Suzuki (1987). Polar lipids were determined by two-dimensional TLC with spraying 5% ethanolic molybdophospholic acid, ninhydrin, Dittmer and Lester reagent, anisaldehyde reagent, and Dragendorff reagent as described by Lechevalier et al. (1977) and Minnikin et al. (1984). Cellular fatty acids were methylated with 5% HCl-methanol (Komagata and Suzuki, 1987). Methylated esters were determined using the MIDI microbial identification system (Microbial ID; Agilent Technologies Inc., CA, USA) according to the method described by Sasser (1990).

### 2.8. Accession numbers

Strain Q-1 was deposited in the Japan Collection of Microorganisms at RIKEN BioResource Research Center (RIKEN-BRC JCM, Ibaraki, Japan) and Laboratorium voor Microbiologie, Universiteit Gent at Belgian Coordinated Collections of Microorganisms (BCCM/LMG, Gent, Belgium) under the culture collection accession numbers JCM 17846*^T^* and LMG 28992*^T^*, respectively. The genome sequence of strain Q-1 was deposited in the DDBJ/EMBL/GenBank nucleotide sequence database under accession number BKCN00000000.

## 3. Results

### 3.1. Growth characterization of strain Q-1

**FIGURE 1:**
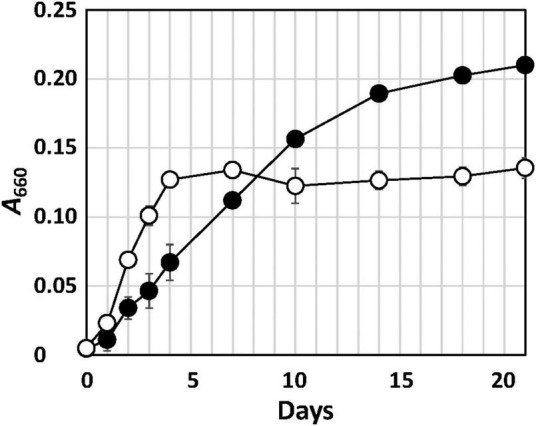
Growth curve of strain Q-1 in the presence or absence of nitrate. Filled circle: YSw medium, and open circle: YSw medium containing 10 mM nitrate. Data represent mean values (*n* = 3), with standard deviations less than 19.4% of the corresponding mean values.

Strain Q-1 grew in the presence of yeast extract under aerobic conditions in air, but not under anaerobic conditions in a N_2_-CO_2_ (4:1, vol/vol) atmosphere. Under aerobic conditions, the growth rate of strain Q-1 was enhanced using nitrate (10 mM) as an electron acceptor in YSw medium in the presence of 0.1% (wt/vol) yeast extract as an electron donor and carbon source (Figure 1). The growth yield was lower than that in the absence of nitrate, which may be due to the production of 3.5 ± 0.2 mM nitrite determined as the sole metabolic product from nitrate. The ammonium concentration was 0.7-0.8 mM, which was an initial concentration in the medium prior to inoculation. Under anaerobic conditions, strain Q-1 did not grow using nitrate (10 mM) as an electron acceptor in YSw medium. Under both aerobic and anaerobic conditions, sulfate (10 mM), sulfite (2 mM), thiosulfate (5 mM), elemental sulfur (1%, wt/vol), nitrite (2 mM), ferric oxide (2 mM), and ferric chloride (2 mM) were not used as alternative electron acceptors in the presence of yeast extract. Under aerobic conditions, strain Q-1 oxidized iodide (2 mM) in the presence of 0.1% (wt/vol) yeast extract, but the turbidity in this culture was almost the same compared with that cultured in the absence of iodide (0.236 ± 0.005 and 0.210 ± 0.004 in turbidity at 660 nm, respectively), indicating that iodide did not support growth as the sole electron donor for chemolithotrophic growth. Hydrogen was not also used as an electron donor in the presence of yeast extract (0.041 ± 0.003 in turbidity at 660 nm).

### 3.2. Corrosion of Fe^0^ by strain Q-1 and *Iodidimonas* strains

The Fe^0^-corroding activities of strain Q-1 and two *Iodidimonas* strains were tested in the corrosion test medium under various culture conditions. Strain Q-1, *I. muriae* C-3*^T^*, and *I. gelatinilytica* Hi-2*^T^* oxidized iodide in the corrosion test medium supplemented with a Fe^0^ foil and 10 mM iodide in the presence of 5% oxygen (vol/vol) (Table 1). Bright yellow deposits that appear to be iron oxide accumulated at the bottom of the culture media, but the iron surface did not lose its shine. The amount of oxidized iron in these cultures was 0.4-1.8 times that of the aseptic control. All three strains did not oxidize the Fe^0^ foil and iodide under N_2_-CO_2_ [4:1 (vol/vol)].

**TABLE 1:** Fe^0^ corrosion and iodide oxidation to iodine in cultures of strain Q-1 and *Iodidimonas* strains.

Species	Strain	Oxygen*	Iron oxidized (mM)	Iodide oxidized (mM)
Unidentified bacterium	Q-1	5%	0.4 ± 0.01	5.5 ± 0.3
		none	0.2 ± 0.03	0.9 ± 0.05
*Iodidimonas muriae*	C-3^T^	5%	1.2 ± 0.01	4.0 ± 0.5
		none	0.2 ± 0.02	0.3 ± 0.03
*Iodidimonas gelatinilytica*	Hi-2^T^	5%	1.7 ± 0.54	3.0 ± 0.3
		none	0.2 ± 0.04	0.6 ± 0.08
Aseptic control		5%	0.9 ± 0.12	0.1 ± 0.05
		none	0.2 ± 0.03	0.1 ± 0.05

Strain Q-1 and each of two *Iodidimonas* strains were grown at 30°C for 28 days under an atmosphere of N_2_-CO_2_-O_2_ (15:4:1) or N_2_-CO_2_ (4:1) in a 70 ml serum bottle containing an Fe^0^ foil immersed in 20 ml of corrosion test medium. Concentrations of oxidized iron and iodide were determined in each culture on day 28. Data are mean values and standard deviation (*n* = 3). *Concentrations of oxygen of 5% and none indicate an atmosphere of N_2_-CO_2_-O_2_ (15:4:1) and an atmosphere of N_2_-CO_2_ (4:1), respectively. An aseptic control indicates an axenic culture of uninoculated *Iodidimonas* strains.

The Fe^0^ foil in the culture of strain Q-1 was oxidized under aerobic conditions when the corrosion test medium was supplemented with 10 mM nitrate as an electron acceptor and 0.05% (wt/vol) yeast extract as a carbon source (Table 2). The amount of oxidized iron in strain Q-1 cultures in the presence of nitrate and yeast extract was approximately six times higher than that of the aseptic control. The surface of the Fe^0^ foil in this culture turned black, and bright yellow deposits accumulated at the bottom of the culture fluids. Strain Q-1 did not oxidize the Fe^0^ foil in the absence of nitrate under aerobic conditions and in the presence of nitrate under anaerobic conditions where air was replaced by N_2_-CO_2_ [4:1 (vol/vol)]. *I. muriae* C-3*^T^* and *I. gelatinilytica* Hi-2*^T^* did not oxidize the Fe^0^ foil under any of the culture conditions described above (Table 2).

**TABLE 2:** Fe^0^ corrosion and nitrate reduction to nitrite and ammonium in cultures of strain Q-1 and *Iodidimonas* strains.

Species	Strain	Oxygen*	Nitrate	Iron oxidized (mM)	Nitrate reduced (mM)
Unidentified bacterium	Q-1	5%	10 mM	6.0 ± 0.4	5.3 ± 0.1
		5%	none	0.4 ± 0.1	–
		none	10 mM	0.6 ± 0.04	1.1 ± 0.2
*Iodidimonas muriae*	C-3^T^	5%	10 mM	0.3 ± 0.1	0.5 ± 0.2
		5%	none	0.3 ± 0.1	–
		none	10 mM	0.3 ± 0.2	0.2 ± 0.1
*Iodidimonas gelatinilytica*	Hi-2^T^	5%	10 mM	0.3 ± 0.1	0.4 ± 0.3
		5%	none	0.3 ± 0.1	–
		none	10 mM	0.2 ± 0.04	0.4 ± 0.6
Aseptic control		5%	10 mM	1.0 ± 0.03	0 ± 0.4
		5%	none	0.8 ± 0.1	–
		none	10 mM	0.1 ± 0.01	0.1 ± 0.2

Strain Q-1 and each of two *Iodidimonas* strains were grown at 30°C for 28 days under an atmosphere of N_2_-CO_2_-O_2_ (15:4:1) or N_2_-CO_2_ (4:1) in a 70 ml serum bottle containing an Fe^0^ foil immersed in 20 ml of corrosion test medium, or the medium without nitrate. Concentrations of oxidized iron and nitrate were determined in each culture on day 28. Data are mean and standard deviation (*n* = 3). –: Not detected. *Concentrations of oxygen of 5% and none indicate an atmosphere of N_2_-CO_2_-O_2_ (15:4:1) and an atmosphere of N_2_-CO_2_ (4:1), respectively. An aseptic control indicates an axenic culture of uninoculated *Iodidimonas* strains.

**FIGURE 2:**
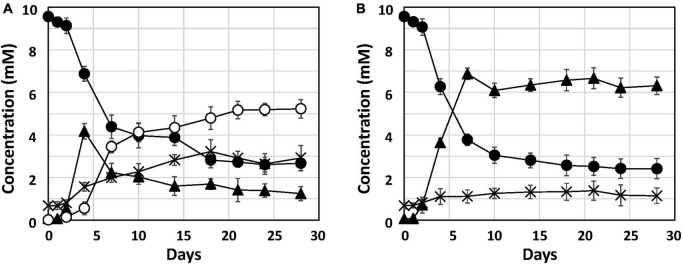
Nitrate reduction and accumulation of nitrite and ammonium in cultures of strain Q-1. Strain Q-1 grew under aerobic conditions of N_2_-CO_2_-O_2_ (15:4:1) in corrosion test medium either in the presence **(A)** or absence **(B)** of Fe^0^ foil. Filled circle: nitrate concentration, filled triangle: nitrite concentration, cross: ammonium concentration, and open circles: oxidized iron concentration. Data represent mean values (*n* = 3), with standard deviations less than 14.3% of the corresponding mean values.

To investigate Fe^0^ corrosion by strain Q-1 with concomitant nitrate reduction, the time course of nitrate, nitrite and ammonium concentrations in strain Q-1 cultures in the presence or absence of Fe^0^ was measured for 28 days. In the presence of Fe^0^, as shown in Figure 2A, The concentration of oxidized iron increased sharply during 4-7 days, followed by a gradual increase. In the presence and absence of Fe^0^ (Figures 2A, B), nitrate concentration decreased sharply during the first 2 to 7 days, followed by gradual decrease. In contrast to the decrease in nitrate concentration, nitrite concentration increased sharply during the first 2–7 days in cultures without Fe^0^. Ammonium concentration increased slightly throughout the cultivation period. In cultures in the presence of Fe^0^, changes in nitrite and ammonium concentrations were different from those in the absence of Fe^0^. Nitrite concentration increased in the presence of Fe^0^ as well as in the absence of Fe^0^, but those with Fe^0^ were highest on day 4, but eventually decreased to about one-fifth of those in the absence of Fe^0^. On the other hand, ammonium concentration in the presence of Fe^0^ gradually increased until the end of the cultivation period, and was about 2.3 times higher than that in the absence of Fe^0^.

### 3.3. Genome analysis of strain Q-1

Previously, the genome sequence of strain Q-1 was determined with 109 contigs using MiSeq (Ehara et al., 2014). To obtain a better quality genome sequence for the genomic characterization and phylogenetic analysis of strain Q-1, the genome sequence of this strain was determined using Ion Torrent PGM. The genome assemblies of 60 contigs for strain Q-1 resulted in a draft genome sequence of 3,071,408 bp with a G + C content of 56.1% (Table 3). A complete set of genes for the pentose phosphate pathway, the TCA cycle, and an almost complete set of genes for glycolysis were encoded in the genome of strain Q-1. However, genes for hexokinase and phosphofructokinase for glycolysis were missing in this genome. This genome contained genes for catalase-peroxidase, superoxide dismutase, cytochrome *c*, and flagellar synthesis, indicating that strain Q-1 was a motile aerobic bacterium. In addition, a complete set of *iox* gene clusters, including *ioxA*, *ioxB*, *ioxC*, *ioxD*, *ioxE*, and *ioxF* genes was encoded (Suzuki et al., 2012; Ehara et al., 2014). Nearly identical gene clusters consisting of a nitrate/nitrite transporter gene, three nitrate reductase subunit genes (*narG*, *narH*, and *narI*), and one nitrate reductase chaperone were also encoded in its genome. Identical genes of two nitrite reductase genes (*nirK* and *nirS*), two nitric oxide reductase subunit genes (*norBC*), nitrous oxide reductase (*nosZ*), three ammonia monooxygenase subunit genes (*amoABC*), and three nitrogenase subunit genes (*nifDKH*) were not encoded in its genome. Hydrogenase genes, including [NiFe]hydrogenase genes possessed in the iron-corroding *Methanococcus maripaludis* strain OS7 (Tsurumaru et al., 2018), were also not found in the genome of strain Q-1.

**TABLE 3:** General characteristics of the genome sequences of strain Q-1, *I. muriae* C-3^T^, and *I. gelatinilytica* Hi-2^T^.

	1	2	3
Contig	60	52	30
Size (bp)	3,071,408	2,995,498	2,848,557
G + C content (%)	56.1	55.6	55.4
Number of protein-coding DNA sequences	3,372	3,171	3,013
Number of rRNA operons (16S-23S-5S)	3	3	3
Number of tRNAs	46	45	47
16S rRNA gene sequence similarity to strain Q-1	100	95.4	96.1
dDDH value to strain Q-1	100	19.1	17.2
ANI values to strain Q-1	100	73.7	73.4

Strains: 1, Strain Q-1; 2, *I. muriae* C-3^T^; 3, *I. gelatinilytica* Hi-2^T^.

**FIGURE 3:**
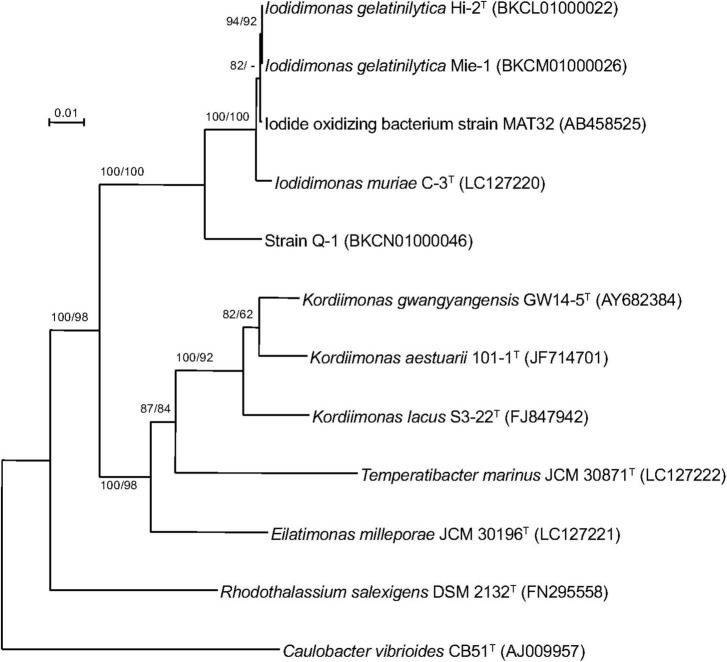
Phylogenetic tree of strain Q-1 and representatives of related species based on 16S rRNA gene sequences. The tree was inferred from an alignment of 1,384 bp of 16S rRNA gene sequences and reconstructed using the neighbor-joining method. Numbers at nodes are bootstrap percentages derived from 1,000 replicates (neighbor-joining/maximum-likelihood). Bar, 0.02 substitutions per nucleotide position. The DDBJ/EMBL/GenBank accession number for 16S rRNA gene sequence is indicated in parentheses.

A single 16S rRNA gene was encoded in the genome of strain Q-1. In the phylogenetic tree of 16S rRNA gene sequences constructed by NJ and ML methods (Figure 3), strain Q-1*^T^* was located close to *I. muriae* C-3*^T^* and *I. gelatinilytica* Hi-2*^T^* and Mie-1 with 95.4, 96.1, and 96.1% sequence similarity, respectively. The topologies of the trees determined by NJ and ML methods were similar. The calculated dDDH and ANI values between strain Q-1*^T^* and *I. muriae* strain C-3*^T^* were 19.1 and 73.7%, respectively (Table 3). Similarly, the calculated dDDH and ANI values between strain Q-1*^T^* and *I. gelatinilytica* strains Hi-2*^T^* and Mie-1 were 17.2-19.3% and 73.4-73.7%, respectively (Table 3).

### 3.4. Taxonomic study of strain Q-1

**FIGURE 4:**
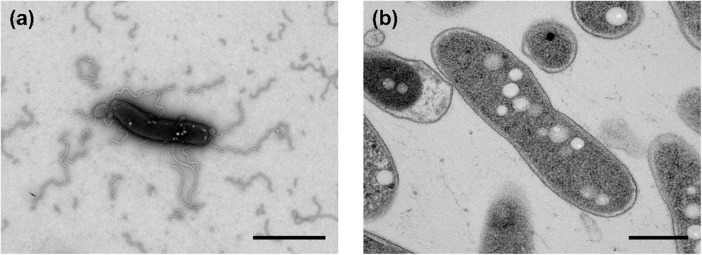
Transmission electron micrographs of cells of strain Q-1. Negatively stained cells and ultrathin sections of the cells are shown in panels **(a)** and **(b)**, respectively. Strain Q-1 was cultured on Marine Agar 2,216 at 30°C for 3 days under air. Bars: 1.0 μm **(a)** and 0.5 μm **(b)**.

The morphological, biochemical and physiological characteristics of strain Q-1, together with *I. muriae* C-3*^T^* and *I. gelatinilytica* Hi-2*^T^*, are summarized in Table 4. The colony appearance of strain Q-1 was circular, convex, opaque, with entire margins, and creamy white in color with a diameter of 0.5-1.5 mm on marine agar after cultivation for 7 days. Cells of strain Q-1 cultured on marine agar 2,216 at 30°C for 2 days were straight rods and approximately 0.3–0.4 μm in width and 1.1–2.0 μm in length (Figure 4a). Vibrational motility was observed by phase-contrast microscopy, but no spore formation was observed. Under light microscopy, the cells were stained Gram-negative. Under transmission electron microscopy, ultrathin sections of whole cells of strain Q-1 stained with uranyl acetate and lead citrate revealed a cytoplasmic membrane and a peptidoglycan layer surrounded by an outer membrane (Figure 4b). Several poorly staining spherical granules were scattered throughout the cells of strain Q-1. Catalase and oxidase reactions were positive. Strain Q-1 was a chemoorganoheterotrophic and aerobic bacterium, and could not grow fermentatively in YSw medium (Iino et al., 2016) containing 10 mM D-glucose under a N_2_-CO_2_ (4:1, vol/vol) atmosphere. The growth temperature for strain Q-1 ranged from 10 to 35°C, with an optimum at 30°C. No growth was observed at 4°C and 40°C. The pH range for growth of strain Q-1 was 4.5–8.5, with an optimum pH range of 7.5. No growth was observed at pH 4.0 and 9.0. Growth occurred in the medium containing 0.5–10% (wt/vol) NaCl, with the optimum being 3% (wt/vol) NaCl. No growth was observed with no NaCl added or with of 11% (wt/vol) NaCl added. Strain Q-1 formed a purple pigment on the marine agar by iodide oxidation. Growth of strain Q-1*^T^* was not enhanced by photoassimilation of organic compounds in YSw medium containing 1.0 g/L yeast extract. Strain Q-1 liquefied gelatin in Marine Broth 2216 (BD, NJ, USA). Black pigment was formed by aesculin hydrolysis on the marine agar. The biochemical characteristics of strain Q-1 using the API 20 NE system (BioMérieux) showed positive results for nitrate reduction, aesculin and gelatin hydrolysis, β-galactosidase, and L-arabinose and D-glucose assimilation, weakly positive for D-maltose assimilation, and negative for reaction to other substrates.

**TABLE 4:** Morphological, biochemical and physiological characteristics that differentiate strain Q-1 from its phylogenetic relatives.

Characteristic	1	2	3
Cell length (μm)	1.1–2.0	1.2–4.4	1.3–3.6
Range in temperature for growth	10–35	4–40	4–40
NaCl range for growth (%)*^a^*:	0.5–10.0	0.5–9.0	0.5–9.0
Nitrate reduction	+	–	–
Hydrolysis of aesculin	+	+	–
Hydrolysis of gelatin	+	–	+
API 20E			
β-Galactosidase	+	–	–
L-Arabinose	+	–	+
D-Maltose	w	+	v

Strains: 1, Strain Q-1; 2, *Iodidimonas muriae*; 3, *Iodidimonas gelatinilytica*. All data were taken from this study. All strains grow with optimum at 30°C, pH 7.5 and 3% (wt/vol) NaCl, are catalase-positive and oxidase-positive, oxidize iodide, did not produce indole and are positive for assimilation of D-glucose in the API20 NE system and negative for the fermentation and the assimilation of D-glucose, arginine dihydrolase, urease and the assimilation of potassium gluconate, malic acid and trisodium citrate in the API20 NE system. +, Positive; w, weakly positive; –, negative; v, variable.

^*a*^Values represent NaCl concentration added to the basal medium containing approximately 0.25% Na^+^ and 0.1% Cl^–^.

The major isoprenoid quinone of strain Q-1 was identified as ubiquinone 10 (UQ-10). The polar lipids of strain Q-1 consisted mainly of phosphatidylethanolamine (PE), phosphatidylglycerol (PG), diphosphatidylglycerol (DPG) and four unidentified aminolipids. The major cellular fatty acids of strain Q-1 were summed feature 8 identified as C_18:1_ω7*c* (29.3%), C_16:1_ω5*c* (25.1%) and C_16:0_ (14.0%) (Table 5).

**TABLE 5:** Cellular fatty acid compositions of strain Q-1 and phylogenetic relatives.

Fatty acid (%)	1	2	3
Saturated straight-chain			
C_12:0_	–	1.7	1.5–6.3
C_14:0_	1.2	1.1	1.0–1.4
C_15:0_	2.0	2.2	1.3–1.6
C_16:0_	14.0	3.0	2.9–4.8
C_17:0_	3.3	3.4	1.4–1.8
C_18:0_	2.0	–	–
Unsaturated straight-chain			
C_16:1_ω5*c*	25.1	17.2	11.5–13.4
C_17:1_ω6*c*	1.5	8.4	4.0–4.2
C_17:1_ω8*c*	1.1	1.5	1.1–1.5
11-methyl C_18:1_ω7*c*	7.0	3.0	4.2–7.5
Hydroxy acids			
C_12:0_ 2-OH	2.1	–	–
C_14:0_ 2-OH	1.2	4.4	2.5–4.2
C_15:0_ 2-OH	–	1.7	–
C_18:1_ 2-OH	7.3	8.4	10.8–14.1
Summed feature*			
3	2.9	2.7	2.6–3.3
8	29.3	38.0	42.0–49.1

Strains: 1, Strain Q-1; 2, *I. muriae* C-3^T^; 3, *I. gelatinilytica* Hi-2^T^. All data were taken from this study. Fatty acids comprising < 1% of the total are not shown.

*The summed feature represents a mixture of fatty acids that cannot be separated by the MIDI system. Summed feature 3 contained C_16:1_ω7*c*, and/or C_16:1_ω6*c*. Summed feature 8 contained C_18:1_ω7*c*.

## 4. Discussion

This study reports the biochemical mechanism of Fe^0^ corrosion by strain Q-1 and its taxonomic characterization. Nitrate reduction was the only characteristic of strain Q-1 that distinguished it from *I. muriae* and *I. gelatinilytica*. In both cultures with or without Fe^0^, nitrate concentration decreased sharply during the first 2–7 days (Figure 2). The reduction curve of nitrate in the presence or absence of Fe^0^ was almost the same. This means that yeast extract was used as an electron donor for the reduction of nitrate as an electron acceptor. Thus, Fe^0^ was not oxidized by coupling with nitrate reduction. Interestingly, in the presence of Fe^0^, nitrite increased until day 4, and decreased from day 7; ammonium increased more than in the absence of Fe^0^. This was demonstrated by nitrite-induced chemical corrosion of Fe^0^ because nitrite oxidizes Fe^0^ in a concentration-dependent manner, as previously shown (Iino et al., 2021b). Evidence that nitrite produced by biological nitrate reduction was the primary causal agent of Fe^0^ corrosion is shown in Figure 2, where nitrite reduction occurred in parallel with Fe^0^ oxidation. Nitrite production was supported by the presence of *narGHI* and the absence of genes for nitrite reductase in the genome sequences of strain Q-1. The amount of 2.57 ± 0.81 mM nitrite decreased between days 4 and 18, and the amount of 2.41 ± 0.69 mM ammonium increased; this was sufficient to produce the observed amounts of 3.78 ± 0.87 mM oxidized iron (3 Fe^0^ + NO_2_^–^ + 8 H^+^ → 3 Fe^2+^ + NH_4_^+^ + 2 H_2_O and 2 Fe^0^ + NO_2_^–^ + 8 H^+^ → 2 Fe^3+^ + NH_4_^+^ + 2 H_2_O). There was no evidence for biotic oxidation of Fe^0^ or Fe^2+^ coupled to nitrate reduction, as it was not enhanced by the presence of Fe^0^ and Fe^2+^ (Figure 2). MIC can be classified into two types: namely chemical MIC (CMIC) and electrical MIC (EMIC), according to the corrosion mechanisms of Fe^0^ (Enning et al., 2012). We propose that strain Q-1 promoted Fe^0^ corrosion mainly by CMIC, as shown in Figure 2, based on the results obtained between days 4 and 18, during which more than 90% of corrosion products were produced.

Iodide oxidation is a unique property of the genus *Iodidimonas* that distinguishes it from the phylogenetically related genera *Eilatimonas*, *Kordiimonas*, *Rhodothalassium*, and *Temperatibacter* (Iino et al., 2016, 2021a). Strain Q-1 also oxidized iodide in common with *I. muriae* C-3*^T^* and *I. gelatinilytica* Hi-2*^T^* and Mie-1 (Iino et al., 2016, 2021a). Wakai et al. (2014) reported that IOB caused iron corrosion in iodine production facilities, and discussed the correlation between iodide oxidation and MIC. Iodide ions are oxidized to molecular iodine by IOB (2I^–^ = I_2_ + 2e^–^), and triiodide ions (I_3_^–^) are solubilized by combining with iodide ions (I_2_ + I^–^ = I_3_^–^). Strain Q-1, *I. muriae* C-3*^T^* and *I. gelatinilytica* Hi-2*^T^* oxidized 3-6 mM iodide. Therefore, 2-3 mM molecular iodine and/or 1-2 mM triiodide ions have been thought to be accumulated by *Iodidimonas* strains. However, in this study, Fe^0^ corrosion was not observed with concomitant iodide oxidation in the corrosion test medium supplemented with Fe^0^ foil and potassium iodide in any of the three strains tested. Unfortunately, Fe^0^ corrosion by strain MAT32 (Wakai et al., 2014) could not be demonstrated because strain MAT32 was not publicly available; this strain may oxidize Fe^0^ by a mechanism different from iodide oxidation.

The phenotypic characterization and phylogenetic localization of Fe^0^-corroding microorganisms are increasingly important for effective MIC prevention and control, as genomic and metagenomic analyses are often applicable for rapid exploration of Fe^0^-corroding microorganisms inhabiting corrosive environments. To clarify the taxonomic position of Fe^0^-corroding strain Q-1, its morphological, biochemical, physiological, chemotaxonomic, and phylogenetic characteristics were determined. The following phenotypic and chemotaxonomic characteristics of strain Q-1 were similar to those of *I. muriae* and *I. gelatinilytica*; e.g., rod-shaped, motile, aerobic, Gram-negative, catalase- and oxidase-positive, mesophilic, neutrophilic, moderately halophilic, iodide oxidation, major menaquione, and polar lipid pattern. In particular, iodide oxidation of strain Q-1 is a unique feature shared with *I. muriae* C-3*^T^* and *I. gelatinilytica* Hi-2*^T^* and Mie-1 (Iino et al., 2016, 2021a), whereas iodide did not support growth as the sole electron donor for chemolithoautotrophic growth. On the other hand, strain Q-1 differed from *I. muriae* and *I. gelatinilytica* in nitrate reduction and aesculin and gelatin hydrolysis as shown in Table 4. In particular, nitrate reduction was supported by the presence of *narGHI* in the genome sequence of strain Q-1, which was absent in the genome sequences of *I. muriae* C-3^T^ and *I. gelatinilytica* Hi-2^T^ and Mie-1. Furthermore, cellular fatty acid profiling showed that the ratio of C_16:0_, C_16:1_ω5*c*, and 11-methyl C_18:1_ω7*c* of strain Q-1 was higher than that of *I. muriae* and *I. gelatinilytica* (Table 5), and the ratio of C_17:1_ω6*c* and summed feature 8 was lower than that of *I. muriae* and *I. gelatinilytica*. In *in silico* DNA–DNA hybridization, the ANI and dDDH values between strain Q-1 and two known *Iodidimonas* species were lower than the threshold of 95% (Yarza et al., 2014) and 70% (Meier-Kothoff et al., 2013) used for prokaryotic species delimitation, respectively. Considering *in silico* DNA–DNA hybridization and the above mentioned differences in phenotypic characteristics and cellular fatty acid composition, it is reasonable to distinguish strain Q-1 from *I. muriae* and *I. gelatinilytica* at the species level. Therefore, a new species with the name *Iodidimonas nitroreducens* sp. nov., was proposed for strain Q-1^T^.

## 5. Conclusion

The MIC of metallic materials imposes a heavy economic burden; therefore, a clear and rapid identification of corrosion-inducing microorganisms is required for MIC prevention and control. This study revealed that *I. nitroreducens* Q-1^T^, as a new species of the genus *Iodidimonas*, induced iron corrosion with concomitant aerobic nitrate reduction. Previously, *Prolixibacter denitrificans* and *Prolixibacter* sp. strain SD074, isolated from an oil well and a crude oil storage tank, have shown to corrode Fe^0^ with concomitant nitrate reduction under anaerobic conditions (Iino et al., 2015, 2021b). Iron corrosion by the nitrate-reducing bacterium *I. nitroreducens* Q-1^T^ has shown that MIC occurs not only under anaerobic conditions but also under aerobic conditions in oil fields and iodine production facilities. In iodine production facilities, IOB including *Iodidimonas* spp. and *Roseovarius* spp. were predominant in the native brine (Wakai et al., 2014). From the perspective of MIC, the genus *Iodidimonas* was divided into two groups: one group was nitrate-reducing *Iodidimonas*, including *I. nitroreducens*, and another group was nitrate-non-reducing *Iodidimonas*, including *I. muriae* and *I. gelatinilytica*. Attention should be paid to the population of nitrate-reducing *Iodidimonas* and the accumulation of nitrite produced by them.

Description of *Iodidimonas nitroreducens* sp. nov.

*Iodidimonas nitroreducens* (nit. ro.re.du’cens. Gr. neut. n. *nitron* niter, nitrate; L. part. adj. *reducens* drawing backward, bringing back to a state or condition; N.L. part. adj. *nitroreducens*, nitrate-reducing).

The following characteristics are given in addition to the species description. Cells are Gram-negative rods, 0.3-0.4 × 1.1-2.0 μm in size, aerobic, motile, and non-sporulating. Colonies are circular, convex, opaque, entire margins, and creamy white in color with 0.5-1.5 mm in diameter on the marine agar. Aerobic and chemoorganoheterotrophic bacteria. Catalase-positive and oxidase-positive. Growth occurs between 10-35°C with an optimum at 30°C. The pH range for growth is 4.5-8.5 with an optimum around 7.5. The NaCl range for growth is 0.5-10.0% (wt/vol), with an optimum at 3% (wt/vol) NaCl. Reduces nitrate to nitrite under air. Sulfate, sulfite, thiosulfate, elemental sulfur, nitrate, nitrite, iron (III) oxide, and iron (III) chloride are not used as sole electron acceptors. Oxidizes iodide on marine agar, whereas iodide did not support the growth as the electron donor. Fermentative growth using D-glucose is not observed. Hydrolyzes aesculin on marine agar. Liquefies gelatin in marine broth. Positive for enzyme reaction of *β-*galactosidase in the API 20 NE system. Not produces indole in the API 20 NE system. Negative for enzyme reaction of arginine dihydrolase and urease in the API 20 NE system. The G + C content of genomic DNA is 56 mol%. The major isoprenoid quinone is Q-10. The major polar lipids are phosphatidylethanolamine, phosphatidylglycerol, diphosphatidylglycerol, and unidentified aminolipids. The major cellular fatty acids are C_18:1_ω7*c*, C_16:1_ω5*c*, and C_16:0_.

The type strain is Q-1^T^ (= JCM 17846^T^ = LMG 28660^T^), which was isolated from iodide-rich brine in Miyazaki, Japan. The G + C content of the genomic DNA of the type strain is 56.1 mol%.

## Data availability statement

The datasets presented in this study can be found in online repositories. The names of the repository/repositories and accession number(s) can be found below: https://www.ncbi.nlm.nih.gov/genbank/, BAYV00000000.

## Author contributions

TI designated the study, performed the experiments and data analysis, and wrote the original draft. KO and MH determined the genome sequence of strain Q-1^T^. MO supervised the research and provided the research funding. SA isolated strain Q-1^T^ and performed the initial characterization. All authors proofread and approved the final version of the manuscript.

## Footnotes

1
http://rast.nmpdr.org

2
http://ggdc.dsmz.de/ggdc.php

3
http://enve-omics.ce.gatech.edu/ani/
