# Corrigendum: Iron corrosion concomitant with nitrate reduction by *Iodidimonas nitroreducens* sp. nov. isolated from iodide-rich brine associated with natural gas

**DOI:** 10.3389/fmicb.2023.1303548

**Published:** 2023-10-10

**Authors:** Takao Iino, Kenshiro Oshima, Masahira Hattori, Moriya Ohkuma, Seigo Amachi

**Affiliations:** ^1^Japan Collection of Microorganisms (JCM), RIKEN BioResource Research Center (RIKEN-BRC), Tsukuba, Japan; ^2^Center for Omics and Bioinformatics, Graduate School of Frontier Sciences, The University of Tokyo, Kashiwa, Japan; ^3^Graduate School of Horticulture, Chiba University, Matsudo, Japan

**Keywords:** microbially influenced corrosion, iron corrosion, nitrate-reduction, iodide oxidation, *Iodidimonas*

In the published article, the accession number for strain Q-1 deposited into Laboratorium voor Microbiologie, Universiteit Gent at Belgian Coordinated Collections of Microorganisms (BCCM/LMG) was incorrect in the Description.

A correction has been made to the section **Conclusion**, as follows:

Description of *Iodidimonas nitroreducens* sp. nov.

*Iodidimonas nitroreducens* (nit. ro.re.du'cens. Gr. neut. n. *nitron* niter, nitrate; L. part. adj. *reducens* drawing backward, bringing back to a state or condition; N.L. part. adj. *nitroreducens*, nitrate-reducing).

The following characteristics are given in addition to the species description. Cells are Gram-negative rods, 0.3–0.4 × 1.1–2.0 mm in size, aerobic, motile, and non-sporulating. Colonies are circular, convex, opaque, entire margins, and creamy white in color with 0.5–1.5 mm in diameter on the marine agar. Aerobic and chemoorganoheterotrophic bacteria. Catalase-positive and oxidase-positive. Growth occurs between 10–35°C with an optimum at 30°C. The pH range for growth is 4.5–8.5 with an optimum around 7.5. The NaCl range for growth is 0.5–10.0% (wt/vol), with an optimum at 3% (wt/vol) NaCl. Reduces nitrate to nitrite under air. Sulfate, sulfite, thiosulfate, elemental sulfur, nitrate, nitrite, iron (III) oxide, and iron (III) chloride are not used as sole electron acceptors. Oxidizes iodide on marine agar, whereas iodide did not support the growth as the electron donor. Fermentative growth using D-glucose is not observed. Hydrolyzes aesculin on marine agar. Liquefies gelatin in marine broth. Positive for enzyme reaction of β-galactosidase in the API 20 NE system. Not produces indole in the API 20 NE system. Negative for enzyme reaction of arginine dihydrolase and urease in the API 20 NE system. The G + C content of genomic DNA is 56 mol%. The major isoprenoid quinone is Q-10. The major polar lipids are phosphatidylethanolamine, phosphatidylglycerol, diphosphatidylglycerol, and unidentified aminolipids. The major cellular fatty acids are C_18:1_ω7*c*, C_16:1_ω5*c*, and C_16:0_.

The type strain is Q-1^T^ (= JCM 17846^T^ = LMG 28992^T^), which was isolated from iodide-rich brine in Miyazaki, Japan. The G + C content of the genomic DNA of the type strain is 56.1 mol%.

The authors apologize for this error and state that this does not change the scientific conclusions of the article in any way.

